# Imipenem resistance in clinical *Escherichia coli* from Qom, Iran

**DOI:** 10.1186/s13104-018-3406-6

**Published:** 2018-05-18

**Authors:** Saeed Shams, Ali Hashemi, Mohammad Esmkhani, Somaye Kermani, Elham Shams, Alessandra Piccirillo

**Affiliations:** 1 Cellular and Molecular Research Center, Qom University of Medical Sciences, Qom, Iran; 2 Department of Microbiology, School of Medicine, Shahid Beheshti University of Medical Sciences, Tehran, Iran; 3 Ali Ebne Abitaleb Hospital, Qom, Iran; 4 Department of Comparative Biomedicine and Food Science, University of Padua, Padua, Italy

**Keywords:** *Escherichia coli*, Imipenem, Metallo-β-lactamase, Resistance

## Abstract

**Objective:**

The emergence of metallo-β-lactamase-producing *Enterobacteriaceae* is a worldwide health concern. In this study, the first evaluation of MBL genes, *bla*_*IMP*_ and *bla*_*VIM*_, in *Escherichia coli* resistant to imipenem isolated from urine and blood specimens in Qom, Iran is described. Three hundred urine and blood specimens were analysed to detect the presence of *E. coli*. Resistance to imipenem and other antimicrobials was determined by disk diffusion and MIC. MBL production was screened using CDDT. PCR was also carried out to determine the presence of *bla*_*IMP*_ and *bla*_*VIM*_ genes in imipenem-resistant isolates.

**Results:**

In total, 160 *E. coli* isolates were collected from March to May 2016. According to disk diffusion, high-level of resistance (20%) to cefotaxime was observed, whereas the lowest (1%) was detected for tetracycline. In addition, five isolates showed resistance to imipenem with a MIC ≥ 4 µg/mL. CDDT test confirmed that five isolates were MBL-producing strains, but no *bla*_*IMP*_ and *bla*_*VIM*_ genes were detected. Results of this study show a very low level of resistance to imipenem in our geographical area.

## Introduction

In recent years, the emergence of antimicrobial resistance in bacteria, mainly *Enterobacteriaceae* and other Gram-negative bacteria, has become a major concern for health systems worldwide [1]. The impact of resistance on cost and length of hospitalization and increase in morbidity and mortality of patients is now obvious. Resistance to carbapenems is considered at high frequency among Gram-negative bacteria, e.g. *Pseudomonas aeruginosa*, *Acinetobacter* spp., and *Enterobacteriaceae* [2–4]. Bacterial β-lactamases can be categorized into four molecular classes based on the amino acid sequence [5]. Metallo-β-lactamases (MBLs) or B class β-lactamases are distinct from the serine β-lactamases (classes A, C, and D) due to a zinc ion(s) in their structure. Except monobactams, these enzymes are able to hydrolyse all β-lactam antibiotics, such as penicillins, cephalosporins and carbapenems. Different types of MBL genes have been described, such as *bla*_IMP_, *bla*_VIM_, *bla*_SPM-1_, *bla*_GIM-1_, *bla*_SIM-1_, *bla*_KMH-1_, *bla*_DIM-1_ and *bla*_NDM-1_ genes [6, 7]. Among these, IMP (imipenemase) and VIM (Verona integron-encoded MBL) types are the most common MBLs that have been recently recognized in *Enterobacteriaceae* [8]. MBLs are encoded both by chromosomal and acquired genes, called resident and acquired MBLs, respectively. Acquired MBLs can spread horizontally via mobile genetic elements among *Enterobacteriaceae* and other Gram-negative bacteria of clinical importance. In addition, studies showed evidence of a broad distribution of MBLs across different geographical areas, and that is considered a serious threat [9, 10]. Thus, although different studies on MBL-producing *Escherichia coli* from some Iranian provinces have been performed, this is the first study carried out in Qom city.

## Main text

### Methods

#### Bacterial isolation and identification

This cross-sectional study was conducted on patients admitted to the *Ali Ebne Abitaleb* Center, Qom, Iran and the Microbiology Department of Qom University of Medical Sciences. During a 3-month period (March to May 2016), 300 blood and urine samples were collected from 300 patients, mean age 40 years, with some common symptoms (e.g., fever, leukocytosis, dysuria, pyuria, and bladder pain), following their consent. Patients with a previous antimicrobial consumption were excluded from the study. Isolation and identification of *E. coli* was performed by a standard procedure. Briefly, blood and urine samples were cultured onto Eosin Methylene Blue agar (Merck, Germany) and MacConkey agar (Merck, Germany) at 37 °C for 24 h. Colonies were confirmed by Gram staining and biochemical testing (e.g., catalase, oxidase, Triple Sugar Iron Agar, Sulfide Indole Motility (SIM), Methyl Red (MR)/Voges-Proskauer (VP), citrate and urease).

#### Antimicrobial susceptibility testing (AST)

Antimicrobial susceptibility of isolates was determined by standard disk diffusion method as recommended by the CLSI [11]. The antimicrobial disks used were imipenem (IMP, 10 µg), ceftazidime (CAZ, 30 µg), amikacin (AK, 30 µg), tobramycin (TOB, 10 µg), gentamicin (GM, 10 µg), tetracycline (TE, 30 µg), ceftriaxone (CRO, 30 μg), norfloxacin (NOR, 10 µg) ciprofloxacin (CP, 5 µg), cefexime (CFM, 5 µg) nalidixic acid (NA, 30 µg), trimethoprim/sulfamethoxazole (SXT, 2.5 µg), amoxicillin (AMX, 25 μg) and cefotaxime (CTX, 30 µg) (MAST, UK). *E. coli* ATCC 25922 was used as a quality control strain.

Minimum inhibitory concentrations (MICs) of imipenem were also determined using the broth microdilution method [11]. Eight different dilutions (256, 128, 64, 32, 16, 8, 4 and 2 μg/mL) were prepared in Mueller–Hinton Broth (Merck, Germany) followed by the transfer of 100 μL into 96-well microtiter plates. Twenty-four hours cultures of *E. coli* colonies were suspended in 0.9% saline at 0.5 McFarland and then diluted (1:20). Finally, 10 μL of the bacterial suspension was added to the wells and microplates were incubated aerobically at 37 °C for 18–24 h. *E. coli* strains with a MIC ≥ 4 µg/mL were defined as resistant strains [11].

#### Phenotypic identification of MBLs

Phenotypic detection of MBLs was evaluated by the combination disk diffusion test (CDDT) using EDTA/imipenem and imipenem disks (MAST, UK) as previously described by Fallah et al. [12].

#### DNA extraction and detection of MBL genes

DNA was extracted from resistant isolates using the boiling method [13]. PCR was performed by using the primers listed in Table 1 and in a total volume of 25 μL composed by 12.5 µL of PCR Master Mix (SinaClon, Iran), 5 µL of extracted DNA, 10 p.m. of each primer synthesized by Bioneer (Korea), and 5.5 µL of DNase/RNase-free sterile water.

**Table 1:** Primers used in this study to detect *bla*_*IMP*_ and *bla*_*VIM*_ genes

Gene	Primer sequences	Name	Size of PCR products (bp)	Reference
*bla*_*VIM*_	5′-GTTTGGTCGCATATCGCAAC-3′	VIM-F	382	[12]
	5′-AATGCGCAGCACCAGGATAG-3′	VIM-R		
*bla*_*IMP*_	5′-GAAGGCGTTTATGTTCATAC-3′	IMP-F	587	
	5′-GTATGTTTCAAGAGTGATGC-3′	IMP-R		

PCR reaction was carried out in a thermocycler (Eppendorf, Hamburg, Germany) as follows: denaturation at 94 °C for 3 min (1 cycle), followed by 35 cycles at 94 °C for 1 min, 55 °C for 1 min, and 72 °C for 2 min and a single, final, elongation step at 72 °C for 10 min. Finally, amplified products were analysed by electrophoresis in a 2% agarose gel run at 95 V for 100 min in 1X TBE containing DNA Safe Stain (SinaClon, Iran) and gels were visualized under UV light.

### Results

**Fig. 1:**
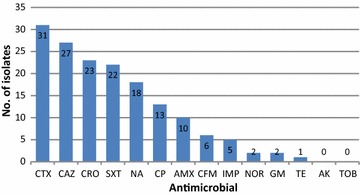
Resistance of *E. coli* isolates (number of isolates)

**Fig. 2:**
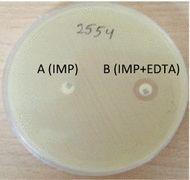
MBL phenotypic test. Disk A; imipenem and disk B; imipenem/EDTA combination. The inhibition zone of B was interpreted as positive

Out of 300 clinical specimens, 160 (53%) were found positive to *E. coli* and all were urine specimen. Sixty percent of strains were isolated from female and 40% from male patients. Disk diffusion test showed that the highest resistance was against cefotaxime (31 isolates, 20%) and ceftazidime (27 isolates, 17%) and the lowest to tetracycline (1 case, 1%). No resistance to amikacin and tobramycin was observed among isolates. Only five isolates (3%) were found resistant to imipenem (Fig. 1). In CDDT assay, all five imipenem-resistant strains were confirmed positive for MBL enzymes (Fig. 2) and they had a MIC ≥ 4 µg/mL. PCR did not detect any *bla*_IMP_ and *bla*_VIM_ genes in MBL-producing strains.

### Discussion

Over the years, the widespread use of antimicrobials has forced the emergence of resistance in pathogenic bacteria, and this has seriously challenged their clinical effectiveness. In the past, carbapenems, mainly imipenem, were considered drugs of the first line in empirical treatment of severe bacterial infections. However, a dramatic increase of resistance to these antimicrobials has being observed in the last years and this can reduce treatment options in the near future [14]. MBLs are effective enzymes in hydrolysing all β-lactams, except for aztreonam. IMP-1 and VIM-1 have been the first enzymes to be discovered in Japan (1990) and in Italy (1999), respectively. Afterward, many variants have been described in other countries. Currently, more than 20 different IMP and VIM types are known [9, 15, 16]. MBL-producing bacteria, *e.g. E. coli*, *Pseudomonas aeruginosa*, *Klebsiella* spp., *Acinetobacter baumannii*, have been reported from different parts of Iran [3, 17–19] and this has become a major threat to public health [20]. *E. coli* is considered an opportunist pathogen and cause of urinary infections, bacteraemia, neonatal meningitis, but the emergence of MBL-producing strains is rapidly growing and threating successful therapy [21]. Therefore, detection of resistance in *E. coli* from different areas is crucial to avoid its spread among bacteria.

In this study, all *E. coli* positive cultures were obtained from urine samples. This finding is in agreement with Ntirenganya et al. [22] who found 55.2% of positive cases related to urine specimens. In our *E. coli* isolates, the highest level of resistance was detected against cefotaxime (20%), ceftazidime (17%), ciprofloxacin (14%) and trimethoprim/sulfamethoxazole (14%). The lowest level of resistance was identified against imipenem (3%), gentamycin and tetracycline (1%), while no resistance to tobramycin and amikacin (0%) was detected. In other Iranian regions, different resistance levels have been reported. Mansouri [23] reported very high resistance rates of *E. coli* to trimethoprim/sulfamethoxazole (93.4%) and amoxicillin (91.4%) in Kerman city, Iran. Unlike our study, the Authors did not report resistance to imipenem. Pouladfar et al. [24] reported two imipenem-resistant urinary *E. coli* (1.9%) in Shiraz, Iran. The full susceptibility of our isolates to tobramycin and amikacin is similar to that obtained in other countries, such as Spain [25]. However, resistance to these antimicrobials has been observed in other studies. For example, Soleimani et al. [26] detected resistance to aminoglycosides among uropathogenic *E. coli* isolated from Tehran, Iran. In addition, they showed that 24.6 and 3.62% of isolates were resistant to tobramycin and amikacin, respectively. Another report from Punjab, Pakistan, showed 59% and 91% of resistance to tobramycin and amikacin, respectively [26, 27].

MIC results were comparable with those obtained at disk diffusion, which showed 3% of isolates resistant to imipenem. A very low level of resistance was observed by Moayednia et al. [28] in Isfahan city, Iran. The Authors detected 0.3% (2/720) of hospital *E. coli* isolates positive for MBL, while no MBL-producing isolates were detected in non-hospital *E. coli*. In contrast to our study, Zeighami et al. [29] did not detect any MBL-positive *E. coli* in Zanjan City, Iran. Besides Iran, the presence of MBL-producing *E. coli* has been reported from other parts of the world. Bora et al. [30] in Nepal indicated a high prevalence of MBLs with 18.98 and 21.08% for *E. coli* and *K. pneumonia*, respectively. Other studies carried out in Korea and India also detected an increasing frequency of MBL positive strains within the *Enterobacteriaceae* family [31, 32].

In this study, no *bla*_*IMP*_ and *bla*_*VIM*_ genes were found, but we cannot exclude that other MBL genes might have been involved in resistance. It is cleared that the resistance to carbapenems may be attributed to outer membrane protein deletion and significantly a decrease in cell permeability can be seen [33]. In study of the Ranjan and co-worker [34], *bla*_*KPC*_ gene could not be detected among isolated *E. coli*, while they were positive for *bla*_*NDM*_ and *bla*_*OXA*-*48*_. Additionally, although phenotypic tests are easy and have an excellent sensitivity, the use of EDTA can give false positive results due to the instability of the bacterial membrane [35, 36]. In agreement with our study, Lee et al. [37] found only one out of three imipenem-resistant *E. coli* isolates as MBL producer and harbouring MBL genes in southern Taiwan. In another study carried out by Peirano et al. [38], the majority of VIM- and IMP-producing *Enterobacteriaceae* were *K. pneumoniae* and only one *E. coli* strain was found positive. A high prevalence of MBL-producing *E. coli* was detected in the study of Nahid et al. [39] in Pakistan. The Authors showed that, out of 145 *E. coli*, 50 (34.48%) were MBL producers and VIM and IMP genes were present in eight and two strains, respectively.

In conclusion, our study is the first description of MBL-producing *E. coli* in Qom, Iran. Overall, strains showed a low resistance level to imipenem and none was found positive for *bla*_*IMP*_ and *bla*_*VIM*_ genes. Therefore, phenotyping assays are recommended for detecting resistance in *E. coli*, as well as a guideline on the prudent use of antimicrobials should be developed for preventing the emergence and spread of resistance.

## Limitations

Due to financial constraints, we could not focus on other genes only.

## Abbreviations

MIC: minimum inhibitory concentration
CDDT: combination disk diffusion test
*E. coli*: *Escherichia coli*
VIM: Verona integron-encoded MBL
PCR: polymerase chain reaction
TBE: Tris/Borate/EDTA
CLSI: Clinical & Laboratory Standards Institute
MBL: metallo-β-lactamases
imp: imipenem
CAZ: ceftazidime
AK: amikacin
TOB: tobramycin
GM: gentamicin
TE: tetracycline
CRO: ceftriaxone
NOR: norfloxacin
CP: ciprofloxacin
CFM: cefexime
NA: nalidixic acid
SXT: trimethoprim/sulfamethoxazole
AMX: amoxicillin
CTX: cefotaxime
